# Cathepsin L as a Driver of Tumour Invasion and a Novel Therapeutic Target in Ovarian Cancer

**DOI:** 10.3390/cancers18091385

**Published:** 2026-04-27

**Authors:** Ielyaa Elshahri, Edward H. B. Ervine, Tala Kamal Musallam, Jawad Alajouz, Muruj Barri, Dmitry A. Zinovkin, Md Zahidul I. Pranjol

**Affiliations:** 1 School of Life Sciences, University of Sussex, Falmer, Brighton BN1 9QG, UKehbervine@gmail.com (E.H.B.E.); mb356@sussex.ac.uk (M.B.); 2 Faculty of Medicine, Al Azhar University, Gaza P.O. Box 1277, Palestine; 3 Department of Pathology, Gomel State Medical University, 246000 Gomel, Belarus; zinovkin_da@gsmu.by

**Keywords:** Cathepsin L, ovarian cancer, tumour microenvironment, extracellular matrix remodelling, metastasis, invasion, angiogenesis, therapeutic targeting

## Abstract

Ovarian cancer is the most lethal gynaecological cancer, largely because it is often diagnosed late and shows strong tumour heterogeneity, therapy resistance, and rapid metastatic spread. A key driver of this aggressive behaviour is the tumour’s ability to reshape its surrounding microenvironment to support invasion, angiogenesis, and escape from treatment. Cathepsin L (CTSL), a lysosomal cysteine protease, has emerged as an important mediator of these processes and is gaining attention as both a prognostic marker and a potential therapeutic target. This review examines the diverse roles of CTSL in ovarian cancer progression, focusing on how its expression, localisation, and extracellular release are altered within the hypoxic and acidic conditions typical of the tumour microenvironment. It also outlines emerging therapeutic strategies aimed at targeting CTSL, including selective inhibitors, multi-cathepsin approaches, CTSL-activated prodrugs and antibody-drug conjugate linkers, and nanomedicine systems designed for tumour-specific delivery. Overall, the evidence highlights CTSL as a central regulator of invasion, angiogenesis, and relapse in ovarian cancer, underscoring its potential as a target for new therapies in aggressive disease.

## Simple Summary

Ovarian cancer is the most lethal gynaecological malignancy because it is often diagnosed late and shows substantial biological diversity, rapid spread, and resistance to treatment. A major reason for this aggressive behaviour is the tumour’s ability to alter its surrounding microenvironment, creating conditions that support invasion, blood vessel formation, and evasion of therapy. Cathepsin L (CTSL), a lysosomal cysteine protease, is emerging as a key player in these processes, with increased expression, altered localisation, and enhanced secretion particularly evident in the hypoxic and acidic conditions typical of ovarian tumours. These changes allow CTSL to promote tissue breakdown, angiogenesis, and metastatic progression, making it both a marker of poor prognosis and a promising therapeutic target. Recent research highlights several CTSL-focused treatment strategies, including selective inhibitors, multi-cathepsin blockade, CTSL-activated prodrugs and antibody–drug conjugate linkers, and nanomedicine approaches designed for tumour-specific delivery. Altogether, current evidence positions CTSL as a central regulator of invasion, relapse, and therapeutic resistance in ovarian cancer, supporting its potential as a target for new treatment approaches in aggressive disease.

## 1. Introduction

Ovarian cancer remains the deadliest of all gynaecological malignancies and a major global health concern, with particularly high incidence and mortality rates in Europe and North America [1,2]. In the UK alone, it ranks as the sixth most common cancer among women [3,4]. The poor prognosis, especially in advanced-stage disease such as high-grade serous carcinoma (HGSC), is largely due to challenges such as chemoresistance, tumour heterogeneity, and enhanced angiogenesis—factors that limit the effectiveness of current treatments. These complexities highlight the urgent need for novel therapeutic targets [2,5,6].

Cathepsins, particularly Cathepsin L (CTSL), have emerged as promising candidates in this context [7,8]. CTSL has been implicated in promoting tumour progression and driving angiogenesis in ovarian cancer [9,10]. In this literature review, we critically evaluate the emerging roles of CTSL in cancer biology, focusing on its involvement in tumour invasion, metastasis, angiogenesis and therapeutic resistance. We also explore recent advances in CTSL-targeted therapies and their potential to improve clinical outcomes.

## 2. Cathepsin L: Structure and Function

CTSL is a ubiquitously expressed member of the papain-like cysteine protease family, encoded in humans by the *CTSL1* gene located on chromosome 9q21–q22 (Uniprot: P07711) [11,12,13]. The enzyme was first isolated in the late 1970s as a lysosomal endopeptidase distinct from cathepsin B [14]. CTSL is synthesised as a ~37 kDa preproenzyme comprising an N-terminal signal peptide (17 aa), a prosegment of approximately 96 amino acids, and a C-terminal catalytic domain (220 aa) [13]. Following co-translational translocation into the endoplasmic reticulum, the signal peptide is removed, and the protein undergoes folding and N-linked glycosylation. Subsequent mannose-6-phosphate tagging in the Golgi apparatus targets pro-cathepsin L to endo-lysosomal compartments via mannose-6-phosphate receptors [15]. Within the acidic lysosomal lumen (pH ~4.5–5.0) [16], proteolytic removal of the prosegment—either through autocatalysis or via other lysosomal cathepsins—yields the mature, active enzyme composed of disulphide-linked heavy (~24 kDa) and light (~5 kDa) chains [13].

X-ray crystallographic analysis reveals that mature CTSL adopts the bilobal “papain” fold that underpins its proteolytic activity. The N-terminal α-helical domain contains the catalytic cysteine residue (Cys25), while the C-terminal β-sheet-rich domain contributes the histidine (His163) and asparagine (Asn187) residues that together form the canonical Cys-His-Asn catalytic triad [17]. In the zymogen, the 96-amino acid prosegment occupies the active-site cleft in a reverse orientation relative to substrate peptides, inserting a conserved glycine residue into the oxyanion hole and sterically blocking both S and S’ subsites. Structurally, the prosegment consists of three α-helices (α_1_–α_3_) stabilised by hydrophobic interactions and multiple salt bridges [18,19]. Its globular N-terminal subdomain interacts with a defined “prosegment-binding loop” (residues ~140–155), while the extended C-terminal arm traverses the substrate-binding groove, forming extensive hydrogen bonds with residues lining the catalytic pocket and thereby maintaining the enzyme in an inactive state [19].

Despite its origin as an intracellular protease, CTSL performs functions extending beyond bulk protein degradation. Under physiological conditions, active CTSL localises predominantly to lysosomes [20], where it degrades endocytosed and autophagic protein cargo, contributing to amino acid recycling and cellular nutrient sensing [21,22]. CTSL also participates in antigen processing by cleaving invariant chain fragments and peptide precursors required for major histocompatibility complex (MHC) class II presentation [23,24]. In addition, CTSL contributes to the regulation of autophagic flux; Dennemarker et al. demonstrated that CTSL deficiency impairs autolysosomal degradation, although compensatory upregulation of cathepsin D can partially mitigate this defect [25]. Beyond lysosomal catabolism, CTSL can process selected precursor hormones and neuropeptides within secretory vesicles [26] and, through alternative translational initiation or limited lysosomal membrane permeabilisation, can transiently localise to the cytosol and nucleus [27], where it cleaves transcriptional regulators such as CDP/Cux, thereby influencing cell cycle progression and transcriptional control [28].

In inflammatory and neoplastic contexts, CTSL is frequently redistributed from lysosomal compartments and secreted into the pericellular milieu, where it contributes to extracellular matrix (ECM) remodelling and tumour progression; this extralysosomal localisation is associated with altered vesicular trafficking, including regulated lysosomal exocytosis and alternative secretory trafficking routes [7,8,27]. Although the neutral pH of the bulk extracellular environment is generally suboptimal for acidophilic cysteine proteases, tumour microenvironments often exhibit localised regions of hypoxia- and V-ATPase-mediated acidification. Plasma membrane-associated vacuolar H^+^-ATPases contribute to pericellular acidification, which creates microdomains favourable for the CTSL’s enzymatic function at the cell surface [7,29]. Under these conditions, CTSL degrades ECM structural components—including collagen IV, fibronectin, laminin, and elastin—thereby facilitating tumour cell migration, invasion and angiogenesis. Such pericellular proteolysis highlights CTSL’s versatile roles beyond lysosomal catabolism, illustrating how cancer cells co-opt its activity to remodel tissue architecture and promote disease progression [30,31,32,33].

Given the potentially deleterious consequences of uncontrolled proteolysis, CTSL expression and activity are tightly regulated at multiple levels. Transcriptionally, CTSL is induced by growth factors such as platelet-derived growth factor (PDGF) and is upregulated downstream of oncogenic signalling pathways, including growth factor receptor-driven signalling, in cancer cells [7,34,35]. Post-translational regulation is mediated by luminal pH and endogenous cysteine protease inhibitors, including cystatins, stefins, and pro-peptides [36]. In addition, vesicular trafficking and subcellular localisation critically govern the spatial distribution of CTSL activity [37,38]. Dysregulation of CTSL expression or mislocalisation contributes to pathological ECM degradation in cancer metastasis, chronic inflammation, and fibrotic diseases, positioning CTSL as an attractive target for small-molecule inhibitors, prodrug strategies, and antibody-based approaches designed to selectively block its proteolytic function within the tumour microenvironment [39].

## 3. Role of Cathepsin L in Tumour Microenvironment

### 3.1. ECM Remodelling and Invasion

**Figure 1:**
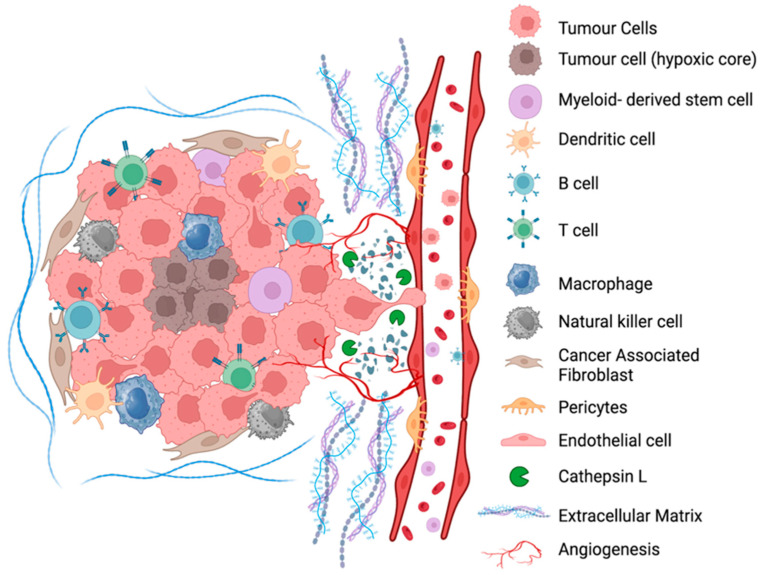
Schematic representation of the tumour microenvironment illustrating the enzymatic function of cathepsin L (CTSL) in extracellular matrix (ECM) degradation. The hypoxic tumour core of a growing tumour releases factors resulting in a pro-inflammatory milieu with cross-communication amongst local cells. Tumour cell-secreted CTSL degrades ECM, forming a pathway that facilitates angiogenesis and tumour cell invasion and migration into circulation to metastasise to distant sites.

CTSL is a key driver of tumour invasion and metastatic dissemination, acting primarily through proteolytic remodelling of the ECM and secondarily through the activation of pro-invasive signalling networks within the tumour microenvironment (Figure 1) [7,40,41]. These processes are initiated and amplified under the hypoxic (~1% O_2_) and acidic (pH ~ 6.8) conditions that typify many solid tumours. Under such stress, highly invasive prostate (PC-3ML) and breast (MDA-MB-231) cancer cells markedly upregulate CTSL secretion via lysosomal exocytosis [7,40]. Sudhan and Siemann demonstrated that hypoxia- and acidosis-induced CTSL release increases extracellular CTSL levels by more than 3-fold, resulting in a 92% increase in invasion and a 74% increase in migration; these effects are almost completely abrogated by the selective CTSL inhibitor KGP94 (10–25 μM; *p* < 0.0001) [40]. Complementary in vivo studies employing CTSL knockdown or pharmacological inhibition further revealed a profound reduction in metastatic burden following intracardiac or tail-vein injection of tumour cells, establishing CTSL as a critical requirement for metastatic dissemination [40,41].

Although CTSL is predominantly a lysosomal cysteine protease under physiological conditions, tumour cells frequently redistribute CTSL to the extracellular and pericellular space, where its activity is preserved within the acidic and hypoxic microenvironments characteristic of solid tumours [40].

Building on this, early biochemical studies established CTSL as a protease capable of directly dismantling basement membrane architecture, demonstrating efficient cleavage of collagen IV, laminin, and fibronectin under mildly acidic conditions compatible with invasive tumour fronts [30]. Subsequent work confirmed that CTSL also degrades elastin and interstitial collagens, reinforcing its capacity to remodel both vascular and stromal ECM compartments relevant to angiogenic invasion [40,42,43].

Consistent with these biochemical properties, once secreted, CTSL remains catalytically active within the pericellular niche, where V-ATPase-mediated proton extrusion maintains a locally acidic microenvironment conducive to cysteine protease activity [29,44]. In this context, CTSL initiates extensive proteolytic remodelling of the ECM by cleaving major structural components, including collagen I and IV, fibronectin, laminin, and elastin, thereby dismantling basement membranes and carving permissive migration tracks through the interstitial stroma [30,45]. CTSL directly activates pro-urokinase-type plasminogen activator (pro-uPA) into enzymatically active uPA, contributing to plasmin generation and downstream proteolysis in the tumour microenvironment [46]. Matrix metalloproteinases are secreted as latent proenzymes and require pro-domain removal through proteolytic cascades involving plasmin and other proteases for activation [47]. Together, these interconnected protease networks accelerate extracellular matrix degradation during tumour invasion and metastasis. In three-dimensional prostate cancer/fibroblast spheroid models, interplay between carcinoma cells and stromal fibroblasts alters basement membrane composition and promotes proteolytic processing of structural ECM proteins, with cancer cell-derived CTSL mediating the generation of biologically active collagen XVIII fragments, including endostatin-containing isoforms, indicating that CTSL-dependent matrix remodelling is potentiated by tumour-stroma interactions in the microenvironment [48].

Concomitant with ECM degradation, CTSL promotes tumour cell plasticity and epithelial–mesenchymal transition (EMT)-associated changes, in part through proteolytic disruption of E-cadherin-mediated adhesion and adherens junction integrity [49,50]. This phenotypic switch endows carcinoma cells with enhanced motility, invasiveness, and resistance to anoikis, thereby coupling matrix remodelling to the acquisition of metastatic competence [51,52]. Additional mechanistic evidence shows that mechanical stress and migratory challenges confer anoikis resistance and metastatic advantage in carcinoma cells [53].

Beyond their direct proteolytic functions, Abl and Arg non-receptor tyrosine kinases activate transcription factors Sp1, Ets1, and NF-κB/p65 in melanoma, which bind to cathepsin promoters and drive increased CTSL transcription and secretion. Abl/Arg-driven invasion in vitro and metastatic spread in vivo are dependent on this enhanced cathepsin secretion, establishing a mechanistic link between oncogenic kinase signalling and protease-mediated tissue remodelling [54].

### 3.2. Angiogenesis

Furthermore, in facilitating tumour cell invasion, CTSL-mediated degradation of stromal barriers permissively enables endothelial cell migration toward the expanding tumour mass, lowering physical constraints on neovascular ingrowth and facilitating vascular access and metastatic dissemination [10,33].

**Figure 2:**
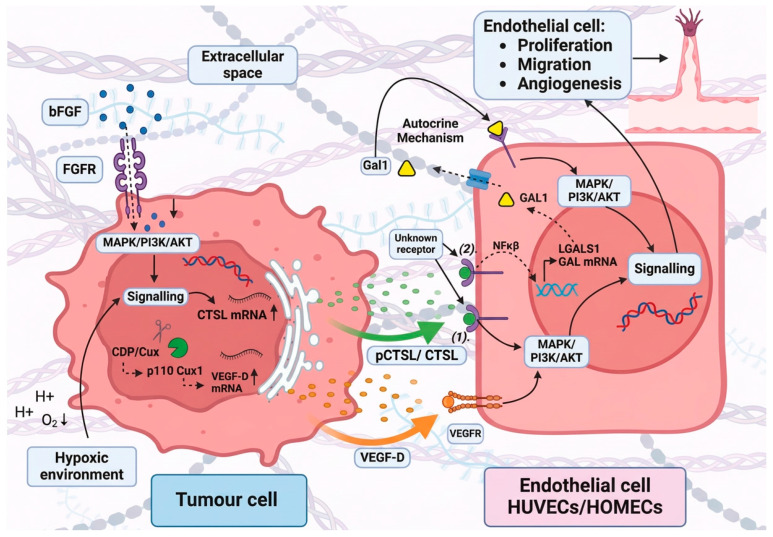
Cathepsin L (CTSL)-induced pro-angiogenic signalling in endothelial cells via intracellular proteolytic and non-proteolytic mechanisms. Growth factors such as basic fibroblast growth factor (bFGF) act on fibroblast growth factor receptors (FGFR) on tumour cells, inducing CTSL synthesis. Tumour cells secrete pro-cathepsin L (pCTSL) and/or active CTSL into the extracellular space in response to hypoxia and growth factor signalling (e.g., bFGF). Extracellular CTSL interacts with a putative cell surface receptor (identity currently unknown), leading to endothelial activation through: (1) direct stimulation of MAPK/ERK1/2 (and potentially PI3K/AKT) signalling pathways, promoting proliferation and migration; and (2) induction of galectin-1 (Gal1) secretion, which acts via an autocrine mechanism to further enhance MAPK signalling and endothelial activation. In parallel, intracellular CTSL can translocate to the nucleus, where it proteolytically processes CDP/Cux to the p110 CUX1 isoform, driving transcription of VEGF-D. Tumour-derived VEGF-D then acts on endothelial cells to promote angiogenesis.

Angiogenesis, the process by which new blood vessels form from existing vasculature, is a critical requirement for tumour growth and metastatic progression [2,55]. Increasing evidence suggests that CTSL is an important regulator of tumour-associated angiogenesis, acting through a combination of direct and indirect mechanisms [33,56]. In line with its established contribution to invasion and metastasis, CTSL can promote a pro-angiogenic microenvironment by supporting endothelial activation and vascular remodelling [7,8,10] (Figure 2). Functionally, genetic suppression of CTSL or pharmacological inhibition using small-molecule compounds (e.g., KGP94) has been shown to reduce tumour-driven angiogenesis in vivo and impair key endothelial processes in vitro, including sprouting, migration, and tube formation [10,33,40,57,58]. Together, these findings position CTSL as a significant driver of pathological angiogenesis and highlight its potential as a therapeutic target in cancer [7,33].

#### 3.2.1. Proteolytic Mechanisms (Extracellular)

A principal mechanism by which CTSL promotes angiogenesis is through proteolytic remodelling of the ECM and vascular basement membranes [33,40,59,60]. Under these conditions, CTSL degrades multiple structural components of the vascular basement membrane and interstitial ECM, including collagen IV, laminin, fibronectin, elastin and fibrillar collagens, thereby removing physical barriers to endothelial invasion and capillary sprout initiation [40,61,62].

In addition to direct matrix degradation, CTSL-initiated proteolysis can liberate ECM-sequestered pro-angiogenic growth factors, including fibroblast growth factors and vascular endothelial growth factors bound to heparan sulphate proteoglycans, further enhancing endothelial recruitment and sprouting [40,63,64].

Functional studies across multiple model systems support a direct link between CTSL-mediated ECM remodelling and angiogenesis [33,60,65]. Genetic or pharmacological disruption of CTSL activity consistently impairs endothelial migration, invasion through ECM substrates, and capillary-like tube formation in vitro, while suppressing tumour-induced neovascularisation in vivo [33]. Quantitative analyses have shown that microenvironmental conditions such as hypoxia or extracellular acidosis markedly increase extracellular CTSL levels, correlating with substantial increases in tumour invasiveness [40], whereas CTSL inhibition reduces endothelial sprouting and angiogenic responses by approximately 50–70% across several assays [33]. These findings collectively indicate that tumour-derived CTSL is functionally required for multiple proteolysis-dependent steps of the angiogenic cascade [33,40,60].

More recent studies have extended these findings to additional tumour contexts, reinforcing the broader relevance of CTSL-dependent angiogenic remodelling [40,60,61,65]. In gastric cancer models, modulation of CTSL expression in tumour cells alters endothelial migration and tubulogenesis in vitro and directly impacts neovascular sprouting and microvessel density in vivo [60]. Reduced CTSL expression is associated with significantly diminished vascularisation and impaired tumour growth, supporting angiogenesis as a rate-limiting process in CTSL-dependent tumour progression [60].

Beyond its role in bulk matrix degradation, CTSL-mediated proteolysis can also influence endothelial behaviour indirectly by activating intracellular migratory programmes [65,66]. CTSL released from growth factor-stimulated cells has been shown to potently enhance endothelial migration via activation of JNK signalling, indicating that CTSL-dependent ECM remodelling can couple physical matrix degradation to pro-migratory signalling once structural constraints are relieved [66].

An additional consequence of CTSL-mediated ECM remodelling is the proteolytic processing of matrix-bound precursors into bioactive fragments that modulate angiogenesis [67,68,69]. Notably, CTSL cleaves collagen XVIII to generate endostatin, a potent endogenous inhibitor of angiogenesis [67]. This cleavage is favoured under mildly acidic conditions characteristic of the tumour microenvironment, indicating that CTSL activity may exert context-dependent effects on angiogenesis depending on the balance between pro-invasive matrix degradation and generation of angiogenic fragments. Together, these findings highlight the context-dependent and bidirectional consequences of CTSL activity within the angiogenic programme [67,68,69].

Consistent with this complexity, genetic deletion studies in the RIP1-Tag2 pancreatic neuroendocrine tumour model revealed that loss of cathepsins B or S reduces angiogenic switching and tumour vascularity, whereas CTSL deletion alone does not alter angiogenic switching or microvessel density despite markedly reducing tumour burden [70,71]. This underscores functional redundancy among cysteine protease networks and indicates that the relative contribution of CTSL to angiogenesis is tumour-type dependent [70,71].

Intracellular nuclear CTSL has also been shown to regulate angiogenic gene expression programmes in other tumour cells, highlighting an additional layer of CTSL-driven angiogenic control [60]. In gastric cancer, CTSL was shown to translocate to the nucleus of tumour cells, where it modulates transcriptional control of angiogenic factor expression [60]. Mechanistically, nuclear CTSL proteolytically processes the transcription factor CDP/Cux (CUX1), converting the full-length p200 form into a truncated p110 isoform with enhanced DNA-binding capacity (Figure 2). This activated CUX1 fragment binds directly to the VEGF-D promoter, driving transcription of VEGF-D, a potent pro-angiogenic cytokine [60]. Silencing CTSL in gastric cancer cells resulted in an approximately 5-fold reduction in VEGF-D mRNA levels and markedly decreased secretion of VEGF-D protein [60]. Functionally, conditioned media from CTSL-deficient tumour cells significantly impaired endothelial tube formation in vitro, while in vivo assays demonstrated a substantial reduction in neovascularisation, including an approximately 3-fold decrease in tumour microvessel density in xenograft models [60]. Restoration of p110 CUX1 expression rescued VEGF-D production and angiogenic activity, confirming that CTSL promotes angiogenesis in this setting through a defined transcriptional axis rather than through extracellular proteolysis [60].

Collectively, these studies establish CTSL-mediated proteolytic remodelling of the ECM as a significant, though non-exclusive, driver of angiogenesis [33,60,70]. By degrading basement membranes, releasing matrix-bound growth factors, and enabling endothelial invasion and migration, CTSL contributes to the creation of a microenvironment permissive for neovascular expansion and tumour progression [30,66].

#### 3.2.2. Non-Proteolytic Mechanisms (Extracellular)

In addition to proteolysis-dependent mechanisms, CTSL also regulates angiogenesis through signalling and transcriptional pathways. Beyond its established role in extracellular matrix degradation, CTSL can influence angiogenesis through a range of non-proteolytic and indirect mechanisms, acting as a signalling molecule and modulator of gene expression within the tumour microenvironment [10,65,66]. These non-canonical functions reveal that CTSL contributes to angiogenic regulation not only by removing physical barriers to vessel growth but also by actively shaping endothelial and tumour cell behaviour through intracellular signalling pathways and transcriptional control.

A compelling example of such non-proteolytic activity has emerged from studies of ovarian cancer metastasis, where CTSL exerts direct pro-angiogenic effects on disease-relevant human omental microvascular endothelial cells (HOMECs) [10]. Recombinant CTSL significantly increased HOMEC proliferation and migration, and the authors concluded that these pro-angiogenic responses are mediated predominantly via a non-proteolytic mechanism [10]. Mechanistically, CTSL-induced activation of the ERK1/2 pathway was implicated in driving these cellular effects, supporting a possible ligand-like mode of action on the omental endothelium [10]. Collectively, these findings support a model in which extracellular CTSL can act as an endothelial-activating signal, promoting angiogenic behaviour independently of its classical matrix-degrading function [10].

CTSL-dependent signalling is not restricted to ERK activation, nor to ovarian cancer alone [10,33,66]. Earlier mechanistic studies demonstrated that CTSL secreted from growth factor-stimulated cells can promote endothelial migration through activation of the JNK pathway, further supporting the concept that CTSL engages conserved MAPK signalling cascades to regulate endothelial motility [66]. Taken together, these findings indicate that CTSL may directly couple tumour-derived signals to endothelial activation via multiple intracellular pathways, thereby reinforcing angiogenic responses without requiring matrix degradation [10,66].

A second mechanistic role is CTSL’s ability to reprogramme endothelial cells to secrete secondary pro-angiogenic mediators, converting a protease signal into a sustained angiogenic circuit. In disease-relevant HOMECs, we showed that recombinant CTSL induces rapid and then amplified Gal1 release: extracellular Gal1 rose ~2-fold at 30 min and peaked at ~5.2-fold at 8 h, returning towards baseline by 24 h [65]. Importantly, the late phase reflected transcriptional input. CTSL increased *LGALS1* mRNA ~1.5-fold at 6 h (*p* < 0.05), and this Gal1 induction was NF-κB dependent, as sulfasalazine reduced CTSL-driven Gal1 secretion from ~4.8-fold to ~0.9-fold (normalised to control) at 8 h. Mechanistically, Gal1-driven endothelial proliferation proceeded via ERK1/2 (not AKT), positioning CTSL as an upstream trigger of an autocrine Gal1-ERK angiogenic loop [65]. These results sharpen the biological interpretation of “CTSL promotes angiogenesis”: beyond permissive ECM degradation, CTSL can act as a stromal signal that rewires endothelial gene expression to amplify angiogenesis through a defined NF-κB-Gal1 axis [65].

Importantly, these indirect mechanisms are not confined to malignant angiogenesis. Genetic ablation studies have shown that CTSL is required for efficient neovascular responses in non-tumour contexts, including post-ischaemic tissue repair, where endothelial progenitor cell invasion and capillary formation are significantly impaired in the absence of CTSL [72]. Consistent with this, CTSL in bone-marrow-derived cells also contributes to pathological ocular neovascularisation [73]. These findings further support the concept that CTSL functions as a broader regulator of angiogenic signalling programmes across physiological and pathological settings [72,73].

Collectively, these studies demonstrate that CTSL exerts multifaceted control over angiogenesis through non-proteolytic and indirect mechanisms. By influencing signalling pathways, including MAPK and NF-κB and modulating transcriptional programmes governing angiogenic factor production, CTSL can orchestrate endothelial activation and vascular expansion across multiple tumour types (Figure 2). These non-canonical roles underscore the complexity of CTSL function within the tumour microenvironment and highlight why therapeutic strategies targeting CTSL must consider not only its protease activity but also its signalling and gene-regulatory functions.

### 3.3. Immune and Stromal Crosstalk

In addition to tumour cell-intrinsic and endothelial mechanisms, CTSL activity is further shaped by interactions with immune and stromal components of the tumour microenvironment. CTSL’s pro-angiogenic output is further amplified by reciprocal signalling between tumour cells, myeloid infiltrates and the stromal vasculature, creating a microenvironment in which proteolysis and paracrine growth programmes reinforce one another [50,60,65,74,75]. Tumour-associated macrophages (TAMs) are a particularly relevant CTSL source because they accumulate at invasive margins, precisely where matrix remodelling and vessel sprouting are coordinated [50,76]. In gastric cancer, Xiao et al. demonstrated strong CTSL co-localisation with the macrophage marker CD68 in patient tissue, consistent with TAMs as a major CTSL reservoir, and showed that macrophage-derived CTSL functionally drives EMT-linked invasion in tumour cells. Co-culture of gastric cancer cells with CTSL-silenced THP-1 macrophages significantly reduced migration and invasion and altered EMT protein profiles. Moreover, co-injection experiments showed that tumours formed with CTSL-knockdown macrophages were significantly smaller than controls, accompanied by clear differences in EMT-associated markers by immunohistochemistry [50]. These data are informative in an angiogenesis context because they establish a macrophage-to-tumour paracrine axis in which macrophage-derived CTSL is not a passive biomarker but an active driver of invasive, hypoxia-associated phenotypes that typically co-evolve with pathological neovascularisation [10,33,50,77,78].

Functionally, myeloid cysteine cathepsin activity is a validated lever for the angiogenic switch. In the RIP1-Tag2 pancreatic neuroendocrine tumour model, Gocheva et al. showed that IL-4-driven alternative activation induces high cysteine cathepsin activity in TAMs in vivo and that TAM-supplied cathepsins, particularly cathepsins B and S, are causally required for robust tumour neovascularisation and growth [75]. Although CTSL was not the dominant effector highlighted in that model, the study is foundational because it demonstrates, using genetic loss-of-function and functional vascular readouts, that myeloid cathepsins sit upstream of tumour angiogenesis, supporting a broader framework in which TAM-derived CTSL contributes to an angiogenic protease network when present within TAM-rich, perivascular niches [75].

CTSL-rich myeloid states also reshape angiogenesis indirectly by sculpting the immune contexture of the tumour microenvironment [79]. Tumour-conditioned dendritic cells (DCs) can adopt tolerogenic phenotypes characterised by elevated secretion of CTSL and other cathepsins, accompanied by increased expression of immunosuppressive enzymes such as IDO1; proteomic profiling of melanoma-conditioned DCs identified a cathepsin-enriched secretome and high cysteine protease activity in tumour-associated DC3-like populations [79]. This matters for angiogenesis because cathepsin-rich immune secretomes do not merely suppress T-cell priming; they also remodel ECM and tune chemokine availability, thereby biasing leukocyte recruitment towards myeloid-dominant, vessel-permissive niches [75,79,80]. Consistent with this, protease-dependent processing of chemokines can erode gradients that support cytotoxic T-cell trafficking while favouring pro-angiogenic inflammatory recruitment, reinforcing a microenvironment in which immune suppression and angiogenesis are mechanistically coupled rather than parallel phenomena [80,81,82].

## 4. Clinical Relevance and Dysregulation of CTSL in Ovarian Cancer and Other Malignancies

### 4.1. CTSL in Ovarian Cancer

The clinical relevance of CTSL dysregulation is underscored by its strong association with aggressive disease and poor patient outcomes. In epithelial ovarian cancer, Zhang et al. reported significantly higher CTSL expression in malignant tumours than in benign and normal ovarian tissues, with elevated CTSL associated with metastatic features, including lymph node involvement, and shorter survival on Kaplan-Meier analysis [83]. Consistent with this tumour overexpression, the same study demonstrated increased serum CTSL concentrations in ovarian cancer patients compared with benign and healthy controls, with higher circulating CTSL associated with poorer overall survival [83].

Ovarian cancer provides the most direct demonstration of this mechanism-to-phenotype link. In HGSC, CTSL expression is markedly elevated within the niche, particularly in tumour cells and microvascular endothelium, where angiogenic remodelling is required to support transcoelomic dissemination [10,65,84]. Functional studies using HOMECs show that tumour-derived CTSL robustly enhances endothelial proliferation, migration, and tubulogenesis, even under VEGF blockade, establishing CTSL as a non-canonical angiogenic stimulus [10,84]. Consistent with this, elevated expression of pro-angiogenic proteases, including CTSL, was observed in patient omental and mesothelial tissues within a highly vascularised metastatic niche [85].

Importantly, these endothelial effects translate to patient tissues: omental metastases display significantly increased microvessel density and vessel area compared with non-malignant omentum, and vascular expansion correlates strongly with CTSL-responsive pro-angiogenic mediators such as galectin-1 within the endothelium [65,86]. Together, these findings support a model in which ovarian cancer exploits CTSL-dependent stromal and endothelial reprogramming to induce a permissive, VEGF-independent angiogenic microenvironment at metastatic sites.

Emerging evidence further indicates that CTSL may exert non-canonical nuclear functions in ovarian cancer. Recent work demonstrates that drug-induced nuclear translocation of CTSL (nCTSL) promotes DNA damage response signalling, enhances cell cycle arrest and apoptosis, and sensitises ovarian cancer models to PARP inhibition, highlighting a previously underappreciated role for CTSL in therapy response and suggesting potential biomarker and combinatorial treatment applications [87].

### 4.2. CTSL in Other Cancers

Evidence from other malignancies reinforces the generality and translational relevance of this axis. In breast cancer, both genetic silencing and pharmacological inhibition of CTSL (notably with the extracellularly active inhibitor KGP94) significantly suppress tumour-induced neovascularisation in vivo, accompanied by reductions in endothelial invasion, sprouting, and tube formation [33]. These vascular effects occur independently of tumour cell proliferation, underscoring a direct role for CTSL in shaping angiogenic responses [33].

In gastric cancer, CTSL expression correlates with increased microvessel density and poor clinical outcomes, and mechanistic studies demonstrate that CTSL activity drives production of VEGF-family ligands via transcriptional reprogramming, linking protease activity to sustained angiogenic signalling [60]. Similar associations between high CTSL levels, vascular invasion and aggressive disease have been reported in colorectal cancer [56]. In melanoma and pancreatic cancer, elevated CTSL has been linked to active modulation of tumour vascularisation [75,88].

Serum CTSL activity has likewise been reported to be elevated in colorectal cancer patients relative to healthy controls and proposed as a candidate biomarker for post-operative follow-up [89]. Beyond ovarian and colorectal cancer, consistent associations between elevated CTSL expression and aggressive clinical features have also been identified in other malignancies; in breast cancer, high CTSL levels correlate inversely with hormone receptor status and independently predict reduced relapse-free and overall survival, with similar patterns of elevated expression linked to more advanced disease observed across multiple solid tumour types [7,90,91].

Clinically, elevated CTSL expression has been linked to aggressive tumour behaviour and angiogenic phenotypes, with upregulation frequently observed in patient tumour samples and associated with poorer outcomes such as relapse and metastasis, particularly in breast cancer [90,91].

Although ovarian cancer provides the most mechanistically resolved model, aberrant CTSL expression is a recurring feature across a wide range of epithelial malignancies, where it often associates with aggressive tumour behaviour and adverse clinical outcomes [83,90,92]. Elevated CTSL levels have been documented in breast, colorectal, gastric, pancreatic, prostate and hepatocellular carcinomas, frequently correlating with advanced stage, increased metastatic burden, and reduced patient survival [41,60,90,92,93,94]. Importantly, CTSL overexpression is observed not only within tumour cells but also within stromal compartments, including tumour-associated macrophages and endothelial cells, underscoring its role in shaping the tumour microenvironment [50,60,93]. Across these cancers, dysregulation of CTSL is often accompanied by loss of endogenous cysteine protease inhibitors, shifting the protease inhibitor balance towards excessive extracellular proteolysis [95]. Together, these clinical and expression data support the concept that CTSL upregulation represents a common oncogenic adaptation in epithelial tumours rather than a tumour-type-restricted phenomenon.

### 4.3. Preclinical Functional Evidence

Crucially, preclinical inhibition of CTSL consistently produces anti-angiogenic and anti-metastatic effects across these models [10,61,75,96]. Suppression of CTSL activity, whether through genetic knockdown, neutralisation strategies, or small-molecule inhibitors, leads to reduced tumour-induced neovascularisation, impaired endothelial function, and diminished metastatic outgrowth in vivo [33,40,97]. These findings highlight an important therapeutic implication: targeting CTSL may simultaneously disrupt tumour invasion and the vascular support required for metastatic colonisation [33,97]. While functional redundancy among cysteine cathepsins and context-dependent effects necessitate careful therapeutic design, the weight of evidence positions CTSL as a critical node in the angiogenic network of the tumour microenvironment [33,70,97].

Functionally, experimental studies across multiple cancer models demonstrate that CTSL contributes directly to malignant progression by promoting invasion, metastasis, and vascular remodelling [40,41]. Genetic or pharmacological suppression of CTSL consistently reduces tumour invasiveness, impairs endothelial migration and sprouting, and limits metastatic outgrowth in vivo, effects that are frequently independent of primary tumour cell proliferation [41,60]. These findings reinforce the notion that CTSL acts as an active driver of tumour progression rather than a passive biomarker of disease severity [40].

Collectively, these mechanistic and clinical insights position CTSL-mediated ECM remodelling as an initiating event that links tumour microenvironmental stress to invasion, EMT, permissive angiogenic remodelling, and metastatic dissemination. This central role has driven substantial interest in therapeutic targeting of CTSL: an I29 propeptide derived from *Calotropis procera* reversibly inhibits CTSL with a K_i_ of ≈1.38 nM and significantly suppresses migration and invasion by more than 60% in vitro of MDA-MB-231 breast cancer cells [96]. In parallel, machine-learning-guided virtual screening approaches have identified candidate inhibitors such as ZINC4097985 with predicted high affinity for the CTSL active site [49]. Emerging nanotechnology-based delivery approaches, including gold- and graphene-based nanoparticles, are being explored and developed to inhibit CTSL within the tumour microenvironment and have been proposed as promising anti-metastatic strategies in preclinical research [61].

### 4.4. Clinical and Translational Implications

Taken together, across multiple experimental systems, CTSL activity translates into clear and clinically meaningful angiogenic phenotypes across diverse tumour types [33,60,65,84,98,99]. In both patient material and experimental models, elevated CTSL consistently associates with increased tumour vascularisation, aggressive growth, and adverse outcomes, positioning CTSL as a biologically relevant driver rather than a passive biomarker of angiogenesis [33,60,65,84].

In summary, CTSL biology matters for tumour angiogenesis because it converts protease activity into durable vascular expansion, acting across extracellular, stromal, and endothelial compartments [65,84,100,101]. This integrated role explains why CTSL inhibition yields vascular and metastatic suppression and supports its consideration as a complementary anti-angiogenic target, particularly in tumours that escape VEGF-centred therapies [33,84,97]. NICE guidance now incorporates biomarker-driven maintenance strategies in advanced ovarian cancer, including PARP inhibitor-based approaches such as niraparib and the biomarker-selected combination of olaparib with bevacizumab [102,103]. However, despite these advances, relapse remains common, underscoring the need to identify additional tumour vulnerabilities that act upstream of dissemination and disease recurrence.

From a clinical perspective, the convergence of correlative patient data and functional preclinical evidence positions CTSL as a biologically significant node within the tumour microenvironment, integrating ECM remodelling, stromal-tumour crosstalk, and angiogenic support [41,60,75,95]. This integrated role provides a strong rationale for targeting CTSL as a therapeutic strategy, particularly in aggressive epithelial cancers where conventional treatments, including VEGF-centred anti-angiogenic approaches, show limited durability [41,55].

### 4.5. CTSL in Therapeutic Resistance and Treatment Response

Therapy resistance remains a central clinical obstacle in ovarian cancer, particularly in the recurrent setting where response durability to platinum-taxane therapy is limited. Current evidence implicates Cathepsin L (CTSL) in treatment response, although ovarian-specific functional data most directly support a role in paclitaxel resistance rather than platinum resistance [9,104]. CTSL is overexpressed in epithelial ovarian cancer and in resistant models, with paclitaxel-resistant SKOV3/TAX cells exhibiting higher CTSL levels than parental cells [9,104]. Functional studies show that CTSL knockdown enhances paclitaxel-induced apoptosis while reducing proliferation, migration and invasion, indicating an active role in maintaining a resistant phenotype [9,104]. These findings align with CTSL’s broader role in promoting tumour cell plasticity and survival through processes including ECM remodelling, EMT, angiogenesis and stromal crosstalk. In this context, CTSL likely contributes to a tumour state less susceptible to therapy-induced cell death. However, CTSL is not yet established as a validated biomarker of platinum resistance in ovarian cancer, and current evidence should be interpreted as mechanistically supportive rather than clinically definitive [9,104].

Evidence from other malignancies further supports a direct role for CTSL in therapy resistance [105,106,107,108,109]. CTSL inhibition has been shown to reverse or prevent resistance by stabilising intracellular drug targets [106]. In BRCA1-deficient breast cancer, CTSL-mediated degradation of 53BP1 alters DNA repair, enabling resistance-associated adaptations [105]. CTSL upregulation has also been linked to cisplatin and paclitaxel resistance via EMT in lung cancer [107], gefitinib resistance in non-small cell lung cancer [108], and chemoresistance in neuroblastoma through regulation of drug efflux, apoptosis and autophagy pathways [109].

Collectively, these data support CTSL as a mediator of therapy resistance through convergent mechanisms involving proteolysis, EMT, survival signalling and DNA damage response adaptation. While ovarian-specific evidence is currently strongest for paclitaxel response, the broader literature suggests CTSL may influence resistance across multiple therapeutic contexts. This provides a rationale for exploring CTSL-targeted strategies in combination with existing therapies while highlighting the need for further ovarian-specific validation.

## 5. Therapeutic Strategies Targeting Cathepsin L

### 5.1. Small-Molecular Inhibitors and Neutral Compound Inhibitors

A growing body of preclinical work supports CTSL as a tractable therapeutic vulnerability in aggressive cancers, with pharmacological inhibition consistently attenuating invasion, metastatic dissemination and, in selected contexts, angiogenic and therapy-response phenotypes [33,40,100,110,111,112,113]. Although no CTSL-selective inhibitor has yet achieved clinical approval, multiple inhibitor classes, ranging from targeted small molecules to broader cysteine-cathepsin blockade and nature-derived scaffolds, provide convergent evidence that suppressing CTSL activity can meaningfully disrupt tumour progression [33,40,96,100,110,111,112,113,114].

The most widely characterised selective CTSL inhibitor in cancer models is the thiosemicarbazone derivative KGP94 (IC_50_ ≈ 0.19 μM) [40,57,58] (Table 1). Across metastatic breast and prostate systems, KGP94 robustly suppresses the microenvironment-enhanced invasive programme driven by hypoxia and extracellular acidification, conditions that markedly elevate extracellular CTSL via lysosomal exocytosis [40]. Functionally, KGP94 nearly abolishes the associated increases in invasion and migration in vitro and reduces metastatic burden in vivo, with limited cytotoxicity at effective doses—consistent with a primary mechanism of inhibiting pericellular proteolysis rather than non-specific growth suppression [40,41,115]. Complementing this benchmark compound, additional medicinal chemistry efforts have produced newer CTSL-selective small molecules with clear anti-metastatic activity in vivo, reinforcing the feasibility of achieving functional selectivity and therapeutic effect [115].

Despite extensive preclinical validation, clinical translation of CTSL-targeted inhibition remains limited. While several multi-cathepsin inhibitors have demonstrated anti-tumour activity in preclinical models, including compounds such as VBY-825, there is currently a lack of CTSL-selective inhibitors progressing through advanced clinical trials. Broader cathepsin-targeting strategies have reached clinical evaluation in other contexts, although safety and selectivity challenges have constrained their development, highlighting the difficulty of translating protease inhibition into effective cancer therapeutics [112,125].

CTSL inhibition may also enhance treatment response beyond direct anti-invasive activity [106,113]. Recent work demonstrates that pharmacological CTSL blockade can augment immune checkpoint therapy, yield additive tumour suppression while simultaneously mitigating treatment-associated cachexia and improving intratumoural CD8^+^ T-cell infiltration [113].

In parallel, computational discovery strategies are expanding CTSL-targetable chemical space. Machine learning-guided screening of natural product libraries has identified candidate CTSL inhibitors (including ZINC4097985 and ZINC4098355) with stable predicted active-site engagement and favourable in silico drug-like profiles, providing plausible leads for subsequent experimental optimisation [49].

Given redundancy within cysteine-cathepsin networks, broader inhibition strategies remain relevant, particularly for tumours that compensate through parallel proteases [70,100]. The pan-cathepsin inhibitor JPM-OEt provides early proof-of-concept that multi-cathepsin blockade can suppress tumour growth, angiogenesis and invasion in vivo, with further reductions in tumour burden when combined with chemotherapy [100,106,126]. Likewise, the reversible multi-target inhibitor VBY-825 (cathepsins B/L/S/V) reduces tumour burden in vivo [112]. More broadly, inhibition of cysteine cathepsins has been shown to preserve bone integrity and suppress osteolytic and skeletal metastatic processes in multiple cancer models [70,111]. A more intermediate strategy is represented by subset-selective inhibition: ASPER-29, a dual CTSL/cathepsin S inhibitor (IC_50_ ≈ 6.0 and 5.0 μM), suppresses migration and invasion in vitro and markedly reduces distant organ metastasis in vivo, supporting the concept that partial network targeting can retain anti-metastatic efficacy while potentially limiting compensatory escape [110].

Nature-derived and peptide-based inhibitors further broaden the CTSL inhibition landscape [96,127]. A potent CTSL prosegment-based inhibitor, the 129 propeptide from *Calotropis procera*, inhibits CTSL at nanomolar affinity (K_i_ ≈ 1.38 nM) and suppresses tumour cell motility in vitro, offering a compelling specificity-driven alternative to small-molecule warheads. However, clinical translation will hinge on overcoming the delivery and stability constraints intrinsic to protein therapeutics [96]. In addition, an AI-driven screen of plant-derived compounds revealed plumbagin and β-lapachone achieve strong enzymatic CTSL inhibition in vitro and display stable active-site binding in computational analyses; while in vivo CTSL-dependent anti-metastatic validation remains outstanding, these compounds represent plausible starting points for potency and selectivity optimisation [127]. Broader naturally derived chemotypes can suppress cathepsin-associated invasive phenotypes even when CTSL selectivity is incomplete. For example, EGCG inhibits tumour invasion by disrupting invadopodia/ECM-degradative programmes through signalling-level mechanisms rather than direct CTSL-selective enzymology, supporting the concept that biologically tolerated scaffolds can converge on protease-dependent invasion circuitry [32,128,129].

Overall, the evidence base supports a coherent therapeutic narrative: selective CTSL inhibition (exemplified by KGP94 and newer chemotypes) can blunt microenvironment-driven invasion and metastatic dissemination; multi-cathepsin inhibition (JPM-OEt and VBY-825) and subset-selective targeting (ASPER-29) address protease network redundancy; and nature-inspired inhibitors (129 propeptide, quinone scaffolds) provide high-affinity or chemically diverse entry points for further development. Together, these approaches substantiate CTSL inhibition as a credible anti-invasive and anti-metastatic strategy and provide a strong rationale for continued optimisation of selectivity, exposure and delivery, particularly in epithelial tumours where protease-driven ECM remodelling and vascular support underpin aggressive clinical behaviour.

### 5.2. Protease-Activated Prodrugs and CTSL-Cleavable Linkers

An increasingly attractive therapeutic strategy exploits CTSL activity itself as a tumour-selective trigger for drug activation [130,131]. In this approach, cytotoxic agents are rendered inert in circulation through masking with CTSL-cleavable motifs and are selectively unmasked only within CTSL-rich tumour microenvironments or endo-lysosomal compartments [130,131]. By coupling drug release to aberrant protease activity, these systems aim to maximise tumour selectivity while limiting systemic toxicity—an especially compelling concept in epithelial malignancies, including ovarian cancer, where CTSL is frequently upregulated and associated with invasive behaviour and chemoresistance [9,83,130,131].

Proof-of-principle for CTSL-activated prodrugs was provided by the development of dual HDAC/CTSL-activated constructs, developed by Ueki and colleagues, in which cytotoxic payloads are released only following sequential deacetylation and CTSL-mediated proteolysis [130,132]. Optimisation of the CTSL-cleavable element in these systems markedly improved intracellular activation, tumour-restricted cytotoxicity, and in vivo efficacy while preserving tolerability [130]. These studies established that CTSL-responsive prodrugs can dramatically widen the therapeutic window, effectively converting highly toxic agents into tumour-confined therapies [130,132]. Given the established role of CTSL in ovarian cancer aggressiveness and therapy resistance, such strategies have clear translational relevance [9,83,104].

Beyond standalone prodrugs, CTSL-cleavable peptide linkers underpin many contemporary targeted drug delivery platforms, most notably antibody-drug conjugates (ADCs) [131,132]. Although early designs focused primarily on cathepsin B-cleavable motifs, subsequent genetic and biochemical analyses revealed that CTSL contributes substantially to linker cleavage, often acting redundantly with other lysosomal cysteine cathepsins [133,134]. This redundancy is advantageous, ensuring robust payload release even in heterogeneous protease landscapes [133,134]. The commonly used Val-Cit linker exemplifies this principle, with CTSL now recognised as an important contributor to intracellular drug liberation alongside other lysosomal cysteine cathepsins [133,134].

**Figure 3:**
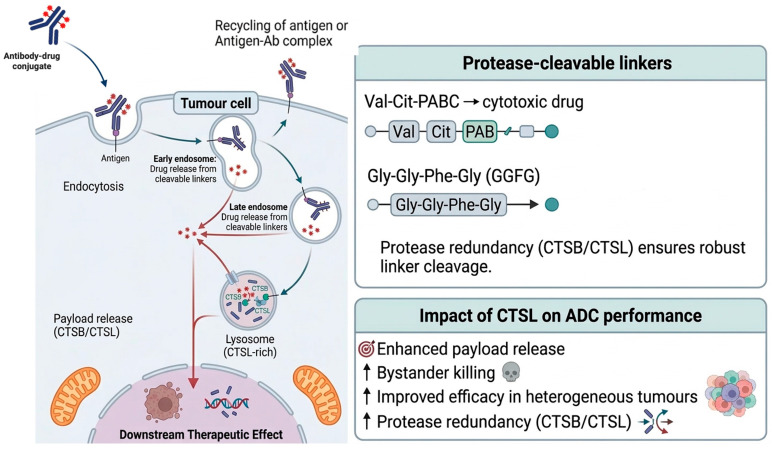
Cathepsin L (CTSL)-dependent activation of antibody-drug conjugates (ADCs). ADCs bind tumour-associated antigens and are internalised via endocytosis, followed by trafficking to lysosomes where protease-cleavable linkers are processed by cysteine cathepsins, including cathepsin B (CTSB) and CTSL. Common linker systems (e.g., Val-Cit-PABC [valine-citrulline-para-aminobenzyl carbamate] and GGFG [glycine-glycine-phenylalanine-glycine]) undergo cathepsin-mediated cleavage; subsequent self-immolation of the PABC spacer enables efficient release of the cytotoxic payload. Protease redundancy (CTSB/CTSL) ensures robust intracellular drug activation. Released payloads induce tumour cell death and may promote bystander killing, enhancing efficacy in antigen-heterogeneous tumours.

The clinical success of trastuzumab deruxtecan (DS-8201a) highlights the therapeutic impact of CTSL-cleavable linker design [131,135,136]. This ADC employs a GGFG-based linker that remains highly stable in circulation yet is efficiently cleaved by lysosomal cathepsins, including CTSL, following tumour cell internalisation (Figure 3) [131]. The resulting release of a membrane-permeable payload enables potent intracellular killing and a pronounced bystander effect, allowing eradication of antigen-heterogeneous tumours [131]. These features underscore how efficient CTSL-mediated linker cleavage can enhance ADC potency, tumour penetration, and therapeutic breadth [131,137].

Importantly, ovarian cancer-directed ADCs increasingly rely on similar cathepsin-cleavable architectures [138,139]. Agents such as folate receptor-α-targeted conjugates employ peptide linkers that undergo cathepsin-dependent cleavage following tumour cell uptake, achieving sustained tumour regression with limited off-target toxicity in preclinical models [138,140,141]. Related ADCs targeting mesothelin and other ovarian cancer antigens likewise exploit CTSL-rich tumour environments to ensure selective intracellular drug release, reinforcing the relevance of CTSL as an activation trigger in this disease context [9,133,142].

In parallel, CTSL-cleavable linkers are being explored beyond classical ADC formats, including small-molecule “smart” prodrugs and hybrid constructs [130,132,137]. Recent work has demonstrated peptide-linked cytotoxic conjugates that undergo efficient CTSL-dependent payload release, validating the feasibility of designing highly optimised CTSL substrates [143,144]. An additional emerging concept involves self-masked CTSL inhibitors, in which potent protease inhibitors are activated only upon CTSL-mediated cleavage, enabling spatially confined suppression of protease activity within tumours [145]. Although still at an early stage, these approaches further highlight the versatility of CTSL-responsive designs [130,132,137,143,145].

Collectively, these studies establish protease-activated prodrugs and CTSL-cleavable linkers as a robust and adaptable therapeutic modality [130,131,132]. By transforming elevated CTSL activity from a driver of tumour progression into a liability, these strategies enable tumour-restricted drug activation, enhanced efficacy and reduced systemic toxicity [130,131]. From optimised dual-activated prodrugs to clinically successful ADCs, the evidence supports CTSL-cleavable systems as a powerful means of exploiting protease dysregulation in cancer [130,131,135,136]. Continued refinement of linker selectivity, pharmacokinetics and delivery, particularly in CTSL-rich epithelial tumours, therefore, represents a well-justified and promising direction for future therapeutic development.

### 5.3. Antibody-Drug Conjugates (ADCs) and CTSL

A major translational application of CTSL-responsive drug release is the antibody-drug conjugate (ADC), where tumour-selective antigen binding enables internalisation and lysosomal trafficking, followed by protease-mediated linker cleavage to liberate a cytotoxic payload [146,147]. Many clinically approved ADCs incorporate protease-cleavable peptide linkers such as Val-Cit-PABC, which were originally designed to be processed by cathepsin B. However, subsequent genetic and biochemical studies have demonstrated that linker cleavage is not exclusively dependent on cathepsin B and that other lysosomal cysteine cathepsins, including CTSL, can contribute to payload release, consistent with recent ADC design strategies that exploit both cathepsin B- and cathepsin L-mediated linker activation [133,143,148,149]. Clinically used Val-Cit-based ADCs, including brentuximab vedotin and polatuzumab vedotin, exemplify this linker class. Evidence that Val-Cit processing is not uniquely dependent on cathepsin B indicates that compensatory cleavage by alternative lysosomal proteases can preserve intracellular payload release across heterogeneous tumour protease environments and highlights CTSL as a relevant contributor to ADC activation [133,148].

This redundancy has important mechanistic and translational implications. CRISPR-based and pharmacological studies have shown that cathepsin B is dispensable for linker cleavage in several ADC systems, with CTSL and related cysteine cathepsins compensating for intracellular processing [133,143]. As a result, linker design is increasingly recognised as a determinant of protease specificity, intracellular stability, and release kinetics [134,143]. The widely used Val-Cit dipeptide linker is efficiently cleaved by multiple cathepsins, whereas tetrapeptide linkers such as Gly-Gly-Phe-Gly (GGFG), employed in deruxtecan-based ADCs, exhibit enhanced lysosomal stability and efficient cleavage by cathepsins, including CTSL [134,143,150]. These linkers are typically coupled to self-immolative spacers such as para-aminobenzyl carbamate (PABC), enabling rapid payload release following proteolysis [149].

The clinical relevance of CTSL-dependent linker cleavage is exemplified by trastuzumab deruxtecan (DS-8201a), a HER2-targeting ADC incorporating a GGFG-based cleavable linker and a topoisomerase I inhibitor payload [131,134]. This construct demonstrates high plasma stability, efficient intracellular activation, and a pronounced bystander effect mediated by membrane-permeable payload diffusion, enabling activity in antigen-heterogeneous tumours [131,151]. Importantly, lysosomal cathepsins, including CTSL, are implicated in the processing of these linkers, reinforcing the functional importance of CTSL in ADC pharmacology [134,150].

Beyond HER2-targeted systems, similar cathepsin-cleavable architectures underpin ADCs targeting clinically relevant antigens in ovarian cancer, including folate receptor-α (FRα) and mesothelin [152,153,154]. FRα-targeted ADCs, such as mirvetuximab soravtansine, and emerging next-generation constructs utilise protease-cleavable linkers to enable tumour-selective payload release following endosomal-lysosomal trafficking [152,155]. Mesothelin-targeting ADCs similarly exploit lysosomal protease activity for intracellular drug activation, supporting the broader applicability of CTSL-dependent processing in ovarian malignancies [156,157]. In these contexts, elevated CTSL expression within ovarian tumours may plausibly influence linker processing and therapeutic response, although direct ovarian cancer-specific evidence remains limited [9].

More broadly, ADC architecture is increasingly being optimised to exploit tumour protease biology [134,158]. Key design parameters include antigen selection, linker chemistry, drug-to-antibody ratio (DAR), and payload properties, all of which influence intracellular trafficking, lysosomal exposure, and protease accessibility [158,159]. Cleavable linker systems that leverage cysteine cathepsin activity offer several advantages, including selective intracellular activation and compatibility with bystander killing mechanisms, although linker stability remains critical to limiting premature systemic payload release [134,159]. These features are particularly relevant in ovarian cancer, where intratumoural heterogeneity and variable antigen expression can limit the ADC efficacy and increase the importance of efficient intracellular processing and bystander activity [159,160].

Collectively, these data establish CTSL not only as a contributor to ADC linker cleavage but as a functionally relevant determinant of intracellular drug release in modern ADC platforms. The increasing use of cathepsin-cleavable linkers, particularly in next-generation DXd-based ADCs, underscores the clinical importance of protease-responsive design and highlights CTSL activity as a potential biomarker of ADC responsiveness and therapeutic efficacy.

### 5.4. Nanomedicine and Targeting Delivery Approaches

Nanomedicine provides a credible route to translate CTSL biology into tumour-selective intervention, either by directly neutralising CTSL activity at invasive fronts or by harnessing CTSL as an enzymatic trigger for conditional drug activation [7,61,161,162,163,164]. This is particularly relevant in ovarian cancer and other epithelial malignancies where CTSL is frequently elevated and associated with metastatic behaviour [7,61,161]. Compared with earlier broad-spectrum protease inhibitors that were constrained by off-target toxicity, nanosystems offer improved opportunities to localise activity to tumours, enhance pharmacokinetics, and restrict systemic exposure through controlled stability and compartment-specific release [7,61,162,164]. Recent advances in cathepsin-targeted nanomedicine further highlight the potential of protease-responsive nanoparticles to enable tumour-selective drug delivery and imaging while also emphasising ongoing challenges in clinical translation, including protease heterogeneity and delivery efficiency [165].

Two dominant CTSL-directed nanomedicine strategies have emerged [7,61]. First, CTSL sequestration (“protease sponge”) platforms, exemplified by graphene oxide nanosheets, aim to physically capture extracellular CTSL under tumour-relevant acidic conditions and thereby suppress protease-driven invasion programmes [61,161]. Second, nanocarrier-based inhibitor delivery approaches use biocompatible carriers (including protein nanocages and functionalised liposomes) to concentrate CTSL blockade at protease-rich tumour sites and within endo-lysosomal compartments, improving intracellular persistence and functional inhibition compared with free inhibitors [61,162,166]. Importantly, these studies also highlight that nanoparticle-protease interactions are not universally inhibitory: certain inorganic nanoparticles have been reported to alter CTSL activity in a surface chemistry-dependent manner, reinforcing that rigorous nanoengineering is essential to achieve reproducible CTSL suppression rather than unintended protease activation [7,61].

In parallel to CTSL inhibition, an orthogonal strategy exploits CTSL as a tumour-selective activation switch [7,61]. Protease-responsive linkers and substrates enable drug payloads to remain inert in circulation yet undergo efficient release following tumour uptake and lysosomal trafficking, a principle validated across targeted conjugate systems [7,164]. Clinically established cathepsin-cleavable motifs (including Val-Cit architectures) demonstrate how linker design can couple intracellular protease activity to selective drug liberation, while related peptide-cleavable polymer-drug systems laid early groundwork for tumour-confined release with reduced systemic toxicity [7,61,133,167]. More recent “carrier-free” prodrug nanoparticles extend this logic further by achieving high drug loading, nanoscale delivery, and endo-lysosomal protease-triggered payload release with improved tolerability in vivo, supporting protease-activated delivery as a broadly scalable strategy even where CTSL is not the dominant activating enzyme [164].

From a translational perspective, these two strategies may be preferentially suited to different tumour contexts [61]. CTSL sequestration approaches are most relevant where extracellular CTSL activity promotes invasion, extracellular matrix remodelling, and metastatic dissemination, suggesting potential utility in highly invasive or peritoneally disseminated ovarian cancers [7,161]. In contrast, nanocarrier-based inhibitor delivery and protease-activated systems are better aligned with settings in which intracellular/endo-lysosomal CTSL activity can be exploited for drug activation or sustained protease inhibition [162,166]. In ovarian cancer, where tumour heterogeneity and peritoneal dissemination are common, these approaches may be complementary: extracellular CTSL targeting may help limit metastatic progression, whereas intracellular protease-responsive delivery systems may enhance tumour-selective cytotoxicity [7,160]. This context-dependent selection highlights the importance of aligning nanomedicine design with tumour protease localisation, disease stage and therapeutic intent [61,162].

Finally, CTSL responsiveness is increasingly being incorporated into advanced precision-design frameworks, including enzyme-gated targeting approaches and multi-trigger “AND-gate” therapeutics [61,168,169]. Dual-enzyme activation systems combining CTSL processing with other tumour-associated biochemical cues have demonstrated enhanced tumour-selective drug deposition and antitumour efficacy in vivo, including in ovarian models, while conditional CTSL-dependent activation has also been integrated into emerging modalities such as activatable degraders [104,130,168,169,170,171]. Collectively, these findings position CTSL-responsive nanomedicine as a versatile therapeutic axis with two complementary translational directions: direct CTSL neutralisation to suppress invasion and metastatic progression and CTSL-activated delivery to confine potent payloads to protease-rich tumour compartments [61,161,164,168]. While challenges remain—including tumour heterogeneity, long-term biodistribution, and the need for predictable nano-protein interactions—the convergence of protease biology with precision delivery strongly supports CTSL-responsive systems as a promising direction for CTSL-high cancers, including ovarian malignancy.

## 6. Conclusions

Recent evidence has consolidated cathepsin L as a pivotal regulator of tumour aggressiveness, acting across multiple hallmarks of cancer progression, including extracellular matrix remodelling, epithelial-mesenchymal transition, angiogenesis, immune reprogramming, and therapeutic resistance. While ovarian cancer remains clinically defined by high rates of late-stage diagnosis, recurrence, and platinum resistance, the mechanistic breadth of CTSL function positions it as more than a correlative biomarker: it is increasingly understood as a functional driver of metastatic competence, particularly within hypoxic and protease-enriched tumour microenvironments. In epithelial malignancies such as ovarian cancer, CTSL overexpression is consistently associated with invasive phenotypes and poorer outcomes, reinforcing its relevance as both a prognostic marker and a rational therapeutic target.

Importantly, the preclinical evidence reviewed here suggests that CTSL inhibition can suppress metastatic dissemination through multimodal mechanisms—including limiting stroma invasion, impairing angiogenic remodelling, and attenuating tumour-supportive macrophage activity while also holding potential to restore sensitivity to standard cytotoxic regimens. This is particularly relevant in ovarian cancer, where clinical strategies already recognise the importance of targeting angiogenesis (for example, bevacizumab-based approaches) and tailoring treatment based on molecular stratification.

Despite these advances, several translational barriers must be resolved before CTSL-directed therapy becomes clinically viable. A major challenge is achieving effective tumour-specific inhibition while avoiding disruption of CTSL’s essential physiological roles in immune regulation, lysosomal homeostasis, and protein turnover. Encouragingly, emerging approaches—such as highly selective small-molecule inhibitors, engineered inhibitory peptides, and tumour-restricted delivery platforms, including protease-cleavable prodrugs, antibody-drug conjugate linkers, and nanocarrier-based systems—offer a credible route to overcome these limitations by confining pharmacological activity to malignant tissues. These innovations shift CTSL targeting from a conceptually attractive strategy to an increasingly actionable therapeutic pathway, potentially in ovarian cancer.

Moving forward, a priority for the field will be the clinical validation of CTSL dependency in ovarian cancer, supported by robust functional biomarkers capable of identifying patients most likely to benefit. Combination strategies may prove particularly valuable, as CTSL inhibition could plausibly synergise with immune checkpoint blockade, anti-angiogenic therapy, or platinum-based chemotherapy by disrupting both physical invasion programmes and tumour-supportive stromal circuits. Finally, comparative and systems-level studies examining protease network redundancy will be critical to determine whether selective CTSL inhibition is sufficient in vivo or whether safe and rational multi-cathepsin approaches are required for durable responses.

In summary, CTSL represents a central node within protease-driven tumour progression, linking microenvironmental stress signals to invasion, vascular remodelling, immune escape, and relapse. The continued convergence of mechanistic cancer biology with structure-guided drug design and precision delivery technologies provides a compelling rationale for CTSL-directed interventions as part of future anti-metastatic treatment strategies. A clinically successful therapy targeting CTSL could ultimately impair a tumour’s capacity to invade, disseminate, and survive treatment—addressing the very processes that drive mortality in late-stage ovarian cancer.

## Figures and Tables

**Table 1:** Inhibitors of cathepsin L in cancers.

Cancer Type	CTSL Inhibition	Outcome	Model System	Refs.
Breast Cancer	Vinyl sulfonate ester derivative (Compound **19**)	Reduces migration	MDA-MB-231	[116]
	1,3-bis(2-fluorobenzoyl)-5-bromobenzenethiosemi-carbazone	70% inhibition of invasion at 10 μM	MDA-MB-231	[117]
	3-benzoylbenzophenone thiosemicarbazone	1- 80% inhibition of migration at 25 μM 2- Tumour growth delay	1- MDA-MB-231 2- C3H mammary carcinoma in CDF1 mice	
	1α,25(OH)_2_D_3_ (vitamin D)	Suppresses CTSL activity; restores 53BP1 levels; increases sensitivity to DNA-damaging strategies resulting in reducing tumour growth	BRCA1-deficient tumour cells	[118]
	3-bromophenyl-3-hydroxyphenyl-ketone thiosemicarbazone (KGP94)	1- 88% inhibition of invasion at 25 μM; 86% inhibition of migration at 25 μM 2- Tumour growth delay	1- MDA-MB-231 2- C3H mammary carcinoma in CDF1 mice	[57,119]
		40% inhibition of migration at 25 μM; 88% inhibition of invasion at 25 μM; 72% attenuation of hypoxia- and acidosis-triggered invasiveness	MDA-MB-231	[40]
Prostate Cancer	3-bromophenyl-2′-fluorophenyl thiosemicarbazone	Reduces DU-145 migration and invasion	DU-145	[120,121]
	1,3-bis(4-fluorobenzoyl)benzene thiosemicarbazone	92% inhibition of invasion at 5 μM	PC-3ML	[117]
	3-bromophenyl-3-hydroxyphenyl-ketone thiosemicarbazone (KGP94)	60% inhibition of migration at 25 μM; 20% inhibition of invasion at 25 μM	DU-145	[57]
		74% inhibition of migration at 25 μM; 88% inhibition of invasion at 25 μM; 50% attenuation of hypoxia- and acidosis-triggered invasiveness	PC-3ML	[40]
		Inhibition of invasion	LNCaP	[7,41]
	Brominated-benzophenone thiosemicarbazone analogue 2	Low cytotoxicity	DU-145	[41,119,120]
Bone Cancer	3-bromophenyl-3-hydroxyphenyl-ketone thiosemicarbazone (KGP94)	Inhibition of migration and invasion	OS-156	[7]
	CLIK-148	1- Reduces tumour-induced hypercalcemia 2- Inhibits bone resorption 3- Reduces bone metastasis	1- LJC-1 inoculation 2- Colon 26 PMF-15 implanted to mouse calvaria 3- A375 tumours in mice	[111]
	iCL (napsul-Ile-Trp-CHO)	Increases sensitivity to chemotherapy	SaOS2	[111]
Neuroblastoma	Z-Phe-Tyr(OtBu)-CHN2 (ZFYD)	Blocked CTSL activity, reduced tumour cell growth	IMR-32, SK-N-SH	[122]
Ovarian Cancer	E-64 (Cathepsin B and L inhibitor)	Decreases spontaneous metastasis	M5076 ovarian sarcoma bearing mice	[123]
Hepatocellular Carcinoma	Tetrahydrobenzofuran derivative	Strong inhibition of CTSL activity	HepG2	[124]

## Data Availability

No new data were created or analysed in this study. Data sharing is not applicable to this article.
